# Comparison of spontaneous eruption and modified closed eruption technique with palatal traction in alignment of impacted maxillary central incisor teeth

**DOI:** 10.1186/s40510-023-00470-7

**Published:** 2023-06-26

**Authors:** Ivo Marek, Anna Janková, Martin Starosta, Michal Novosad, Josef Kučera

**Affiliations:** 1 Department of Orthodontics, Faculty of Medicine and Dentistry, Palacký University Olomouc, Palackeho 700/12, 779 00 Olomouc, Czech Republic; 2 STOMMA Dental Clinic, Tř. 1. máje 3414/11A, 690 02 Břeclav, Czech Republic; 3 Department of Orthodontics, First Faculty of Medicine, Charles University and the General University Hospital in Prague, Kateřinská 32, Prague 2, 12800 Prague, Czech Republic

**Keywords:** Tooth impaction, Central incisor, Closed eruption, Active traction, Spontaneous eruption, Attached gingiva

## Abstract

**Background:**

Central incisor impaction is a rare condition with potentially severe clinical and psychological implications for the patient. Treatment techniques vary according to the pretreatment situation and individual factors. The aim of this study was to compare the esthetic outcomes and treatment times between two different approaches.

**Materials and methods:**

In this retrospective study, thirty-one consecutive patients (13 boys, 18 girls; average age 9.5 ± 2.3 years) with a total of 34 impacted permanent upper central incisors were included in the study. Patients were divided into two groups according to method of treatment. Group A comprised patients in whom spontaneous eruption occurred after space opening (*n* = 12), and Group B comprised patients in whom teeth showed no eruption and required treatment with a modified closed eruption method with palatally oriented traction (*n* = 19). Treatment time and esthetic outcomes were assessed and compared between groups.

**Results:**

The mean treatment time was 22.0 ± 6.7 months, and all teeth were successfully aligned. No statistically significant difference in average treatment time was found between groups in baseline characteristics (*p* > 0.05). The amount of attached gingiva was significantly smaller when compared to contralateral reference teeth in the closed eruption group (Group B; *p* = 0.03). However, no difference in amount of attached gingiva was found between both groups (*p* = 0.26). Additionally, no difference in the clinical crown length was found between groups (*p* = 0.27).

**Conclusion:**

The closed eruption method with palatal traction directed at the peak of the alveolar crest provided results comparable to the physiologic tooth eruption.

## Background

Central incisors rarely become impacted, with an incidence of 0.1–0.5% [1]. The most common causes of central incisor impaction include obstacles in the eruption path (e.g., supernumerary tooth or odontoma), trauma to the deciduous dentition (e.g., intrusive luxation) leading to developmental changes of the permanent tooth (i.e., dilaceration), and a deviated eruption path [2–8].

Typically, the maxillary permanent central incisor begins to erupt around 6 years of age [9, 10]. When physiologic eruption does not occur, the patient should be referred for orthodontic evaluation. Under normal circumstances, the maxillary lateral incisors are the last of the incisors to erupt, appearing approximately one year after the eruption of the adjacent central incisors to complete the anterior dentition. Further changes in dentition do not occur until approximately age 9.5–10 years. As such, there is a two-year period of relative stability known as the “mixed dentition stage”. When both lateral incisors have erupted and one or both of the central incisors are missing, further investigation is warranted [7]. If an impacted tooth is suspected, X-ray imaging is essential for diagnosis (orthopantomogram, OPG), which usually reveals the cause [11, 12]. To obtain a precise diagnosis and to visualize the position and relationship to adjacent anatomical structures, a cone-beam computed tomography (CBCT) is usually indicated [1]. The choice of orthodontic approach for alignment of the impacted tooth into the dental arch should be carefully considered and guided by clinical and radiological examinations [13].

Treatment of an impacted maxillary incisor involves orthodontic space preparation, followed by surgical elimination of any obstacles. In many cases, this may be sufficient to encourage the autonomous eruption of the affected tooth, with periodontal and esthetic parameters approximating those observed with physiologic eruption. However, when the impacted tooth does not spontaneously erupt when the obstacle is removed and sufficient space is available, enhancement of the natural process is required. This entails surgical exposure of the crown of the impacted tooth, bonding of an attachment, and the application of extrusive force between the tooth and an existing labial archwire [7, 14–17]. Choosing the appropriate treatment approach is critical, with many demands being placed on the orthodontist with respect to esthetic outcome, future dental health, and long-term prognosis [17, 18].

**Fig. 1:**
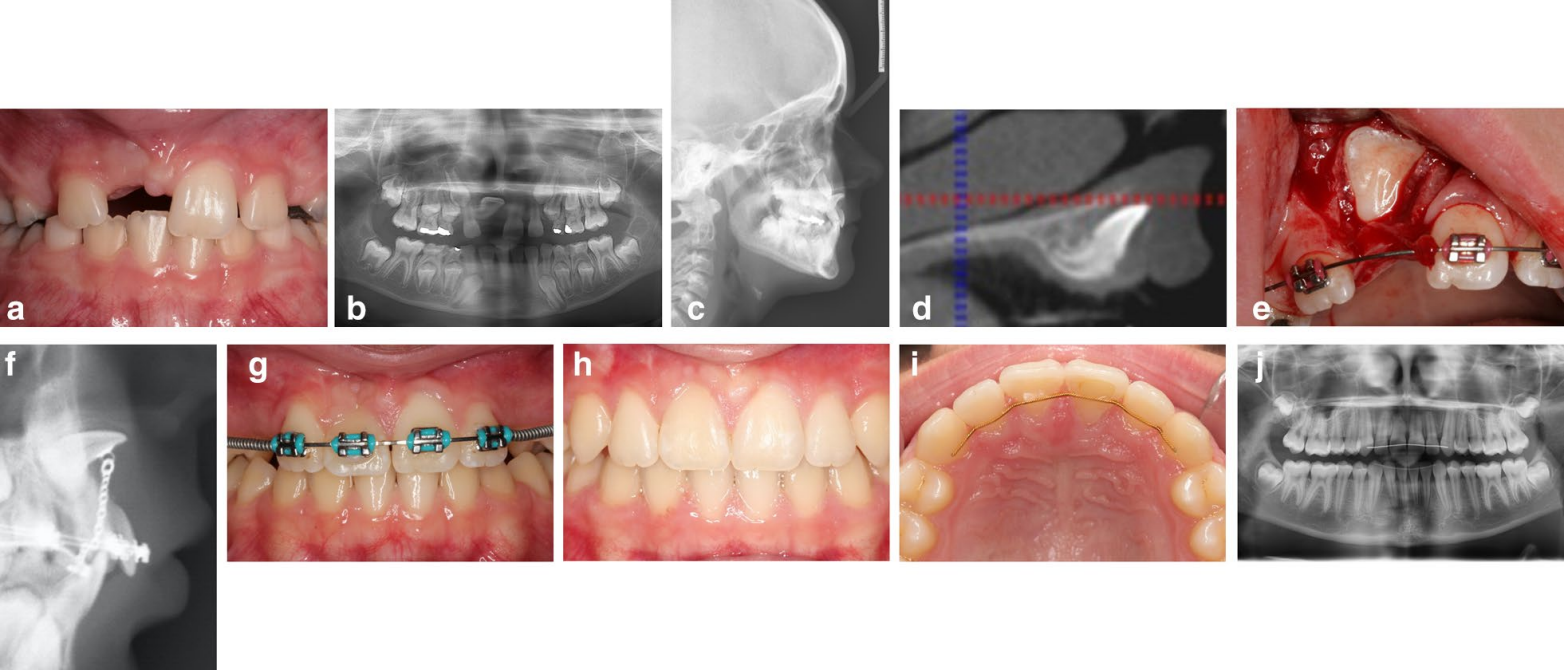
**a**: A seven-year-old patient with complete eruption of both lateral incisors and left central incisor. **b**: Panoramic X-ray image of tooth 11 positioned horizontally with apparent root angulation. **c**: Cephalometric X-ray image of the crown of tooth 11 is inclined in cranial direction. **d**: Cone-beam computed tomography sagittal view showing root dilaceration is evident. **e**: Lifting the mucoperiosteal flap and exposing the palatal surface of the crown of tooth 11 prior to bonding of the attachment. **f**: Cephalometric X-ray image showing the attachment on the palatal surface and chain traction in the palatal direction toward the extension arm of the palatal arch. **g**: Impacted right central incisor aligned into the dental arch. Attached gingiva is apparent due to the eruption of the tooth through the alveolar ridge crest. The crown torque is insufficient. **h**: Ideal gingival margins and equal amount of attached gingiva on teeth 11 and 21 at debonding. **i**: Ideal crown torque of tooth 11 from the occlusal view after debonding. **j**: Panoramic X-ray after treatment demonstrating the same root lengths on teeth 11 and 21

The tooth will almost always erupt, but the outcome is often negatively affected by an insufficient width of attached gingiva and an elongated clinical crown [13]. To overcome potential collateral damage and improve overall prognosis, we present an improved ortho-surgical approach that directs the vertically advancing tooth in a lingual direction, as opposed to the standardly used labial direction [19]. The directional force is applied from a simple spring-drawn or elastic-drawn device which, following a closed exposure procedure, guides the tooth to erupt through the attached gingiva on the crest of the alveolar ridge. This procedure best approximates physiologic spontaneous tooth eruption and as such provides an ideal foundation for long-term esthetics and functionality of the tooth (Fig. 1a–j).

The aim of this study is to compare the outcomes of patients treated with this approach and those treated with spontaneous eruption of impacted teeth following a space opening procedure.

## Material and methods

Thirty-one consecutive patients (13 boys and 18 girls; average age 9.5 ± 2.3 years) with a total of 34 impacted permanent upper central incisors were included in this study. Central incisors were regarded as impacted when one or more of the following features was present: (1) Lateral incisors were already erupted and space reduction for a central incisor had occurred; (2) the roots were dilacerated; (3) the tooth was displaced in an ectopic location as determined by a panoramic radiograph; (4) a supernumerary tooth or other obstruction was present.

All patients underwent orthodontic treatment at STOMMA Dental Clinic (Břeclav, Czech Republic) between 2002 and 2017 performed by the same orthodontist (IM). Surgical uncovering procedures were performed by one of two surgeons at the clinic (MN, MS). All 34 impacted teeth were successfully aligned, and none required extraction. In one patient, a partial obliteration of the pulp was observed. In another, necrosis of the pulp was observed and required a root canal procedure during the retention phase. No pathological changes in the periodontium were found in any patient.

The inclusion criteria for this study were as follows:

1. Completion of orthodontic treatment from initial examination, surgical intervention (if performed), and in retention phase,
2. Availability of full medical records of the treatment course, including all necessary data about the case history, the cause of the tooth impaction, the duration of the orthodontic therapy, and in all Group B cases, the surgical intervention and associated complications;
3. Good-quality panoramic X-rays before and after treatment;
4. A cephalometric radiographic image before and after treatment;
5. Good-quality photographic documentation taken at the beginning, throughout active treatment, after treatment therapy and during the retention phase;
6. Patients in the retention phase for more than 24 months;
7. Patients without clefts, syndromes or other structural malformations.

All patients received orthodontic treatment to open the space for the incisor. This was achieved using a 0.022-inch slot Roth prescription preadjusted fixed appliance (OrthoOrganizers, Carlsbad, USA). An expansion coil spring on rigid stainless steel arches was activated to open sufficient amount of space. The desired space opening was designated as 2 mm wider than the width of the contralateral central incisor. After three months, the situation was examined on panoramic radiograph for any progress toward spontaneous eruption of the impacted tooth.

**Fig. 2:**
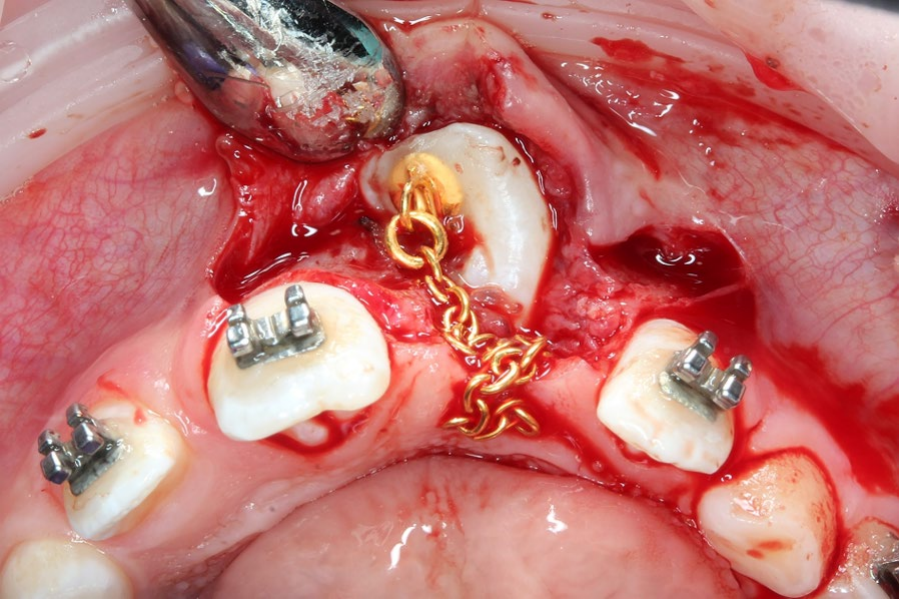
The attachment with the chain is bonded to the palatal surface of the impacted tooth during the surgical procedure

**Fig. 3:**
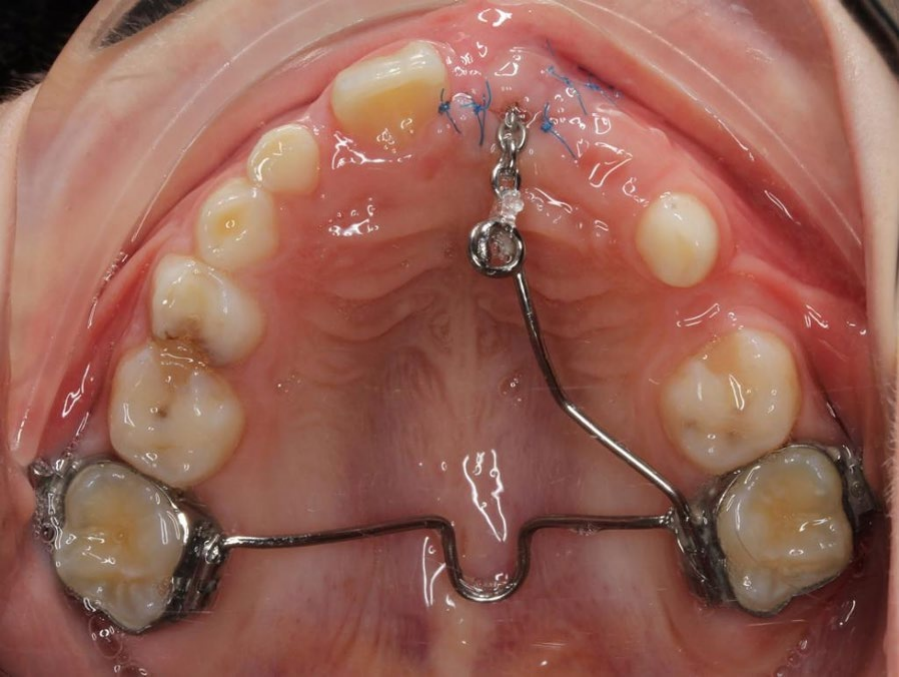
Transpalatal arch with extension arms allows engagement of the metal chain and activation in the palatal direction

Patients were then divided into two groups. For cases in which eruption had occurred spontaneously, the tooth was incorporated into existing orthodontic appliance (Group A; *n* = 12). Cases in which the tooth showed no eruptive progress after three months were subjected to a modified closed eruption surgical exposure procedure (Group B; *n* = 19). This modified procedure was accomplished using the following protocol (18):

1. Reflect the mucoperiosteal flap and uncover the crown by removing bone on the palatal side of the tooth, secure dry field and bond an orthodontic button chain attachment on the palatal surface of the crown using adhesive resin;
2. Replace the pedicle flap to its original position and suture in place;
3. Guide the orthodontic chain under the flap between the sutures in the mid-crestal incision area into the oral cavity to the crest of the alveolar ridge (Fig. 2);
4. Apply an elastic thread to an individually fabricated transpalatal arch with an arm extended to the area of the impacted tooth (Fig. 3);
5. Perform orthodontic activation with a maximum force of 40 g in the palatal direction;
6. Continue step-by-step activation by gradual distal shortening of the arm, redirecting the eruption path of the impacted incisor toward the crest of the alveolar ridge.

In both the groups, a straight wire approach was used with a standard sequence of archwires followed by final individual corrections. In cases of ectopic teeth, additional corrections were made by adding more torque to the archwire or by using modified bracket bonding to achieve the same amount of torque as in the adjacent tooth. A visual control was used to assess tooth position, and root position was checked manually by palpating the root prominence bulge at the alveolar mucosa.

### Patient information

This study was approved by the Ethical committee of the Palacký University Hospital in Olomouc, Czech Republic (Protocol number: EC FNOL 23/21). Informed consent was obtained from the child and parent, or from an adult with parental responsibilities and rights. All procedures were conducted in accordance with the Declaration of Helsinki. The following data were obtained from the orthodontic records: Gender; age at the start of therapy; etiology of impaction (e.g., dilaceration, supernumerary tooth, unknown cause); type of impaction (unilateral or bilateral), date of the beginning of orthodontic therapy (T1; date of bonding of the fixed appliance); date of the end of orthodontic therapy (T2; for interceptive therapy, the date when the fixed appliance was removed, in comprehensive treatment the date when the impacted incisor was leveled and aligned, i.e. day of transition to 0.017 × 0.025-inch stainless steel archwire); and length of treatment (defined as the period from the fitting of the fixed appliance to the end of orthodontic therapy).

### Radiologic assessment

#### Panoramic X-ray analysis

**Fig. 4:**
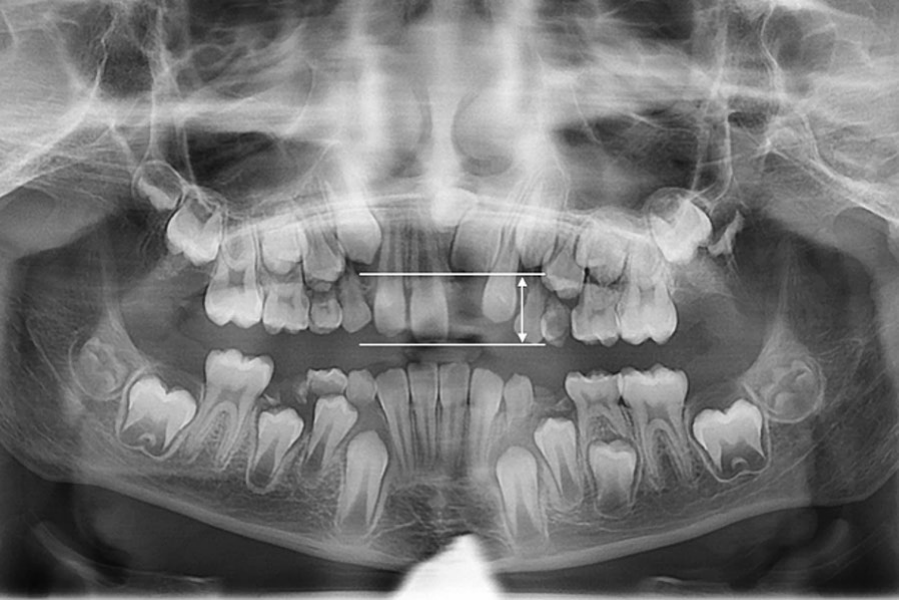
Panoramic X-ray measurement of the degree of impaction from the incisal edge of the impacted tooth to the incisal edge of the neighboring tooth

The degree of impaction was evaluated as previously described by Vermette et al. [20] from the pretreatment panoramic X-ray (T1). A perpendicular line from the incisal edge of the impacted incisor, or from the incisal edge of a physiologically erupted contralateral central incisor was drawn toward the occlusal plane. The length of this line was measured using a distance measurement tool in the software module (Kefalo professional, BelDental, Czech Republic) with a precision of 0.1 mm. This measurement was converted to a nonparametric scale: < 12 mm = simple impaction, 12–15 mm = medium impaction, or > 15 mm = complex impaction (Fig. 4). Root formation was also assessed at T1 and categorized as finished root development (closed apex) or unfinished root development (open apex). In cases where root formation of the impacted tooth could not be established, contralateral tooth was assessed as a reference. Any atypical shape changes in root form (e.g., root dilaceration) were also assessed on panoramic X-ray.

#### Cephalometric image analysis

**Fig. 5:**
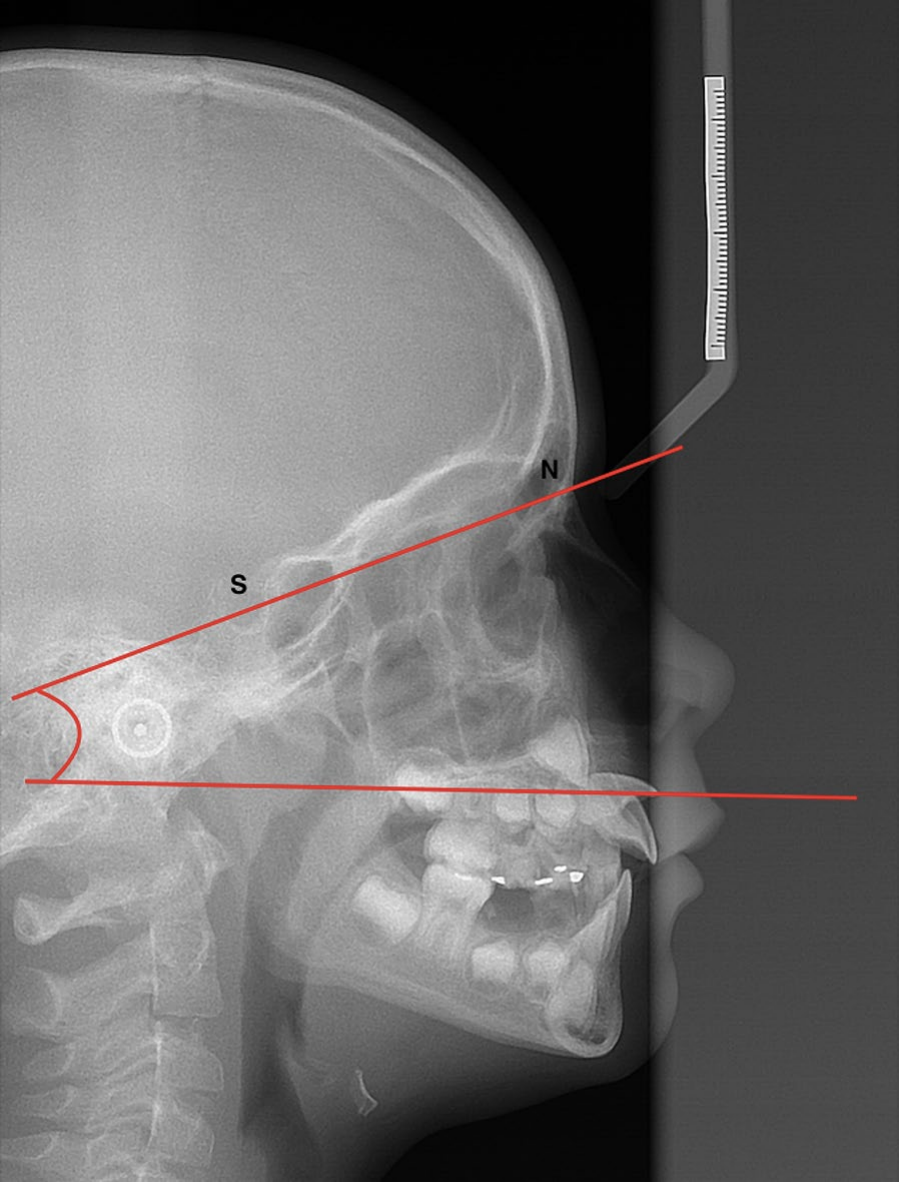
The angle between the long axis of the impacted tooth and the Nasion-Sella reference line (NS Angle)

The angle between the long axis of the impacted tooth and the nasion-sella horizontal reference line (NS-angle) was measured on the cephalometric X-ray. Measurements were made using cephalometric module software (Kefalo professional, BelDental, Czech Republic). According to the size of the angle, the values were divided into three categories: I. 15–42 degrees, II. 43–70 degrees, and III. 71–98 degrees (Fig. 5).

### Intraoral assessment

**Fig. 6:**
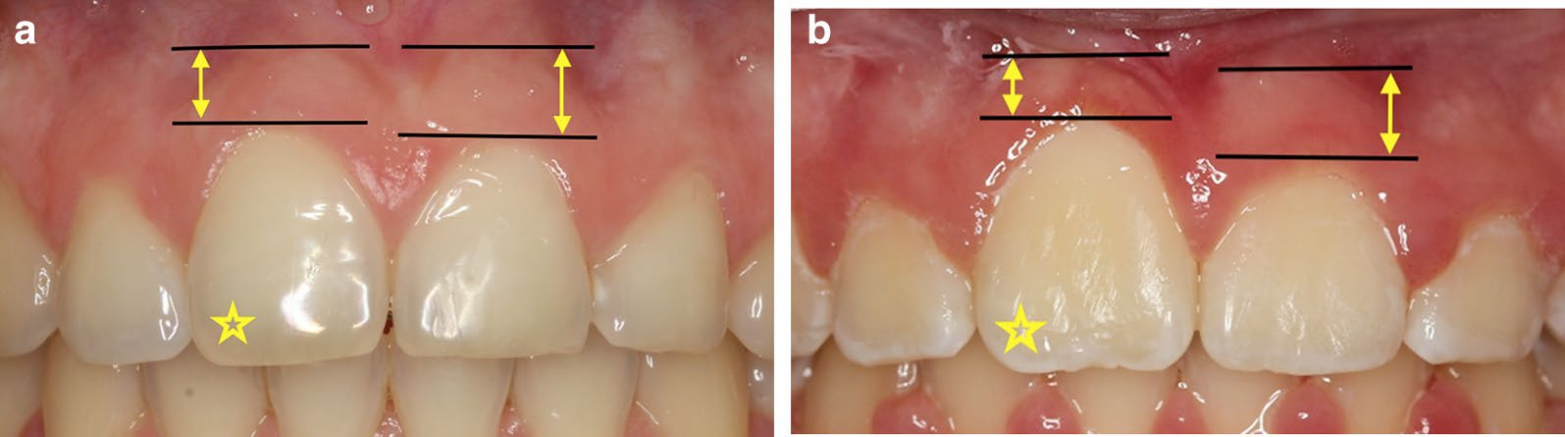
Assessment of the amount of attached gingiva as measured with a caliper (yellow arrow); the impacted tooth is indicated by a yellow asterisk. **a** Attached gingivae on the impacted tooth and the control tooth are similar in size; the gingiva is rated as sufficient. **b** Attached gingivae of the impacted tooth and the control tooth are not similar in size; the gingiva is rated as satisfactory

Intraoral assessment was performed during the retention phase no less than 24 months after orthodontic treatment (T3). With respect to the parameters for which the contralateral, spontaneously erupted central incisor was used as a reference, six teeth in three patients from group B with bilateral central incisor impaction were excluded to form a smaller group (*n* = 28). The following parameters were assessed using a digital caliper with a precision of 0.1 mm (Powerfix, OWIM, Neckalsulm, Germany):

1. The height of the attached gingiva around the impacted tooth and the physiologically erupted contralateral tooth. The attached gingiva was assessed using the following categories of percentage when compared to the contralateral side: not established (0–25%), satisfactory (25–50%), and sufficient (> 50%) (Fig. 6a, b).
2. The length-to-width ratio of the clinical crown of the impacted tooth was compared with the physiologically erupted contralateral incisor. The length (distance from the middle of the incisal edge to the deepest point on the gingival margin) and width (mesiodistal width of the widest crown area) of the clinical crown of the tooth were measured and the ratio was calculated. The results were then categorized as presented in Table 1 as: excellent appearance, adequate appearance, and marred appearance (shortening or lengthening of the clinical crown of the impacted tooth compared with a neighboring tooth).

**Table 1:** Assessment of length-to-width ratios and evaluation of the clinical crowns of the teeth

	Ratio	Evaluation
	10:7.8–8.8	Excellent appearance
		Ideal tooth length-to-width ratio
	10:7.3–7.7	Marred appearance
		Long clinical crown
	10:8.9–9.5	Adequate appearance
		Slightly short crown
	10:9.6–11.5	Marred appearance
		Substantially short crown

### Statistical analysis and error measurement

The data are presented as the mean and standard deviation. The data for both groups were statistically assessed using the Mann–Whitney U test, Fisher’s test, Wilcoxon rank-sum test and Kruskal–Wallis test. All statistical analyses were performed using the IBM SPSS Statistics (Version 23.0, Armonk, NY: IBM Corp., USA) with a *p* value of < 0.05.

The primary investigator reassessed all parameters from ten randomly selected patients to calculate the error of measurement using the Dahlberg error of measurement, interclass correlation coefficient (ICC) and Bland–Altman graphs. Random selection was performed with Excel (Microsoft Inc., USA) using the Rand and Index functions. The Dahlberg error was found to be 0.23 mm for the measurement of the distance of the impacted incisors from the occlusal plane, 0.20° for NS Angle and 0.32 mm for attached gingiva height, the correlation coefficient showed a high level of agreement between first and second measurement (0.991 for attached gingiva height and 0.998 for distance to the occlusal plane). A perfect match was found between the first and second measurements for all categorized parameters (Cohen kappa 1.0). Bland–Altman plots also indicated that the errors were not statistically significant.

## Results

The average patient age at the beginning of treatment was 9.5 ± 2.3 years (boys: 9.8 years; girls: 9.2 years; minimum age 7 years; maximum 16 years). Impaction of the permanent upper central incisor occurred more frequently in girls (*n* = 18; 58%) than in boys (*n* = 13; 42%).

Table 2 shows the comparisons of baseline characteristics of both the groups regarding gender, average age, etiological cause of impaction, degree of impaction, NS angle, and apex maturation. Spontaneous eruption after space opening (Group A) occurred in four girls and eight boys (average age 8.5 years and 10.6 years, respectively) and closed eruption technique was required in 14 girls and 5 boys (average age 9.2 years and 9.4 years, respectively). Of the 31 patients, 16 (51.6%) had impaction on the left, 12 (38.7%) on the right, and three (9.7%) had bilateral impaction of the permanent upper central incisor. Impaction was caused by a supernumerary tooth in 17 teeth (50%; 7 boys and 7 girls with 3 cases of bilateral impaction), and root dilaceration in eight teeth (23.5%; 3 boys and 5 girls). Etiology was unknown in nine patients (26.5%; 3 boys and 6 girls). In group A, the etiological factors included supernumerary tooth in eight cases and root dilaceration in one case. In four cases, no etiological factor could be identified. In group B, nine cases of supernumerary teeth were found and seven cases of root dilaceration. No etiological factor of impaction could be identified in five cases. In both groups, impacted incisors with a wide range of NS angles were found. However, most patients in group A had a favorable (i.e., more vertical) incisor inclination (62%), while in group B most patients had unfavorable (i.e., more horizontal) inclination. In group A, patients with simple impactions are predominant (62%), while in group B most patients had complex impaction (57%). With respect to the apex maturation stage, both groups had a similar distribution.

**Table 2:** Baseline characteristics of both treatment groups regarding gender, age, cause of impaction, NS Angle, degree of impaction and apex maturation

	Group A (*n* = 12)		Group B (*n* = 19)	
	Boys	Girls	Boys	Girls
Gender	8	4	5	14
Average age	10.6	8.5	9.4	9.2

Teeth (*n* = 34)	13		21	
*Impaction cause*				
Supernumerary tooth	8	62%	9	43%
Root dilaceration	1	7%	7	33%
Unknown	4	31%	5	24%
*NS angle*				
15–42°	1	7%	10	48%
43–70°	4	31%	7	33%
71–98°	8	62%	4	19%
*Impaction degree*				
Simple	8	62%	4	19%
Medium	2	15%	5	24%
Complex	3	23%	12	57%
*Apex*				
Closed	10	77%	18	86%
Opened	3	23%	3	14%

Table 3 shows treatment times according to various parameters. The average treatment time was 22.0 ± 6.7 months (minimum 10 months; maximum 35 months). No difference in average treatment time was found between groups according to baseline characteristics such as the orthodontic therapy type, cause of impaction, NS angle, degree of impaction or apex maturation stage (*p* > 0.05). A positive correlation was found between the treatment time and age of the patients for pooled data (*r* = 0.435; *p* = 0.02); however, no significant correlation between these parameters was found in either group (*r* = 0.35; *p* = 0.30 and *r* = 0.35; *p* = 0.17, for group A and group B, respectively).

**Table 3:** Treatment length comparisons according to therapy type, cause of impaction and other baseline characteristics

	*n*	Mean	SD	Min	Max	Median	*p*
*Orthodontic therapy type*							
Spontaneous eruption	13	21.9	6.9	11.0	33.0	23.0	0.99^a^
Closed eruption	21	22.2	6.8	10.0	35.0	23.0	
*Cause of impaction*							
Supernumerary tooth	17	21.4	6.4	11.0	35.0	21.0	0.79^b^
Root dilaceration	8	22.9	5.4	11.0	30.0	24.0	
Not found	9	22.7	8.8	10.0	34.0	22.0	
*NS angle*							
15–42°	11	25.0	7.1	11.0	35.0	25.0	0.21^b^
43–70°	11	20.4	4.5	12.0	26.0	21.0	
71–98°	12	21.0	7.7	10.0	21.5	21.5	
*Degree of impaction*							
Simple	12	20.6	6.5	10.0	33.0	21.0	0.43^b^
Medium	7	21.6	6.7	11.0	31.0	23.0	
Complex	15	23.5	7.2	12.0	35.0	25.0	
*Apex maturation*							
Closed	6	23.6	5.3	15.0	31.0	24.5	0.42^a^
Open	28	21.7	7.0	10.0	35.0	22.0	

^a^Mann–Whitney U test, ^b^Kruskal–Wallis test

Table 4 reports the intergroup comparisons of the attached gingiva assessment. Of the 34 impacted teeth, one (2.9%) had no attached gingiva, six (17.6%) had a satisfactory attached gingiva, and 27 (79.4%) had sufficient attached gingiva. On the adjacent physiologically erupted tooth, the attached gingiva was sufficient in all patients. In group A, where spontaneous eruption occurred after space opening, no difference in attached gingiva was found when compared to that of contralateral physiologically erupted teeth (*p* = 0.18). In group B, where the modified closed eruption technique was performed, a significant difference in attached gingiva was found between impacted teeth and physiologically erupted adjacent teeth (*p* = 0.03). However, no significant difference was found between groups (*p* = 0.26).

**Table 4:** Assessment of the attached gingiva on impacted teeth (*n* = 34)

Attached gingiva		Space opening versus control	Closed eruption versus control
Not established	Number	1	0
	%	7.7	0
Satisfactory	Number	5	1
	%	7.7	23.8
Sufficient	Number	16	11
	%	84.6	76.2
	*p*	0.18^a^	0.03^a^
		0.26^b^	

Comparisons between impacted teeth and control side according to the selected method of treatment (^a^Wilcoxon test) and intergroup comparison (^b^Fisher`s exact test)

Table 5 presents the assessment of esthetic outcome in each group in patients with unilateral impaction (*n* = 28). Three patients with bilateral central incisor impaction were excluded from this analysis, as these cases did not have a contralateral tooth for reference. No significant difference in the esthetic outcome was found between groups (*p* = 0.27).

**Table 5:** Assessment of the clinical crown length (length-to-width ratio) of impacted teeth (*n* = 28) at the end of treatment and comparison with the contralateral control tooth according to the method of treatment (Fisher's exact test)

Clinical crown length		Marred appearance: crown lengthened	Excellent appearance	Adequate appearance:	Marred appearance: crown shortened	Total	*p*
Closed eruption	Number	0	11	2	4	17	0.27
	%	0.0	64.7	11.8	23.5	100.0	
Space opening	Number	2	4	2	3	11	
	%	18.2	36.3	18.2	27.3	100.0	
Total	Number	2	15	4	7	28	
	%	7.1	53.6	14.3	25.0	100.0	

## Discussion

The purpose of this study was to evaluate esthetic and periodontal outcomes of impacted maxillary incisors treated using two different methods: space opening with subsequent spontaneous eruption or a modified closed eruption technique with attachment bonding to the palatal surface and palatally directed orthodontic traction. The results of the current study were also compared to those of similar studies. In general, the assessment of the outcomes of treated impacted permanent upper incisors is challenging; this is likely attributable to the small number of patients included in available studies due to the low prevalence of this dental anomaly [21–26].

Normal physiologic tooth eruption in the developing dentition ensures the natural creation of an ideal attached gingiva as teeth erupt in the middle of the alveolar crest. Conventional wisdom holds that therapeutically attempting to redirect the eruption of an errant tooth through the middle of the crest of the alveolar ridge should achieve the same. Accordingly, the closed eruption method has been recommended as a method of choice because it most closely approximates the physiological process of tooth eruption. This treatment approach also prevents buccal tooth eruption through the alveolar ridge mucosa where the attached gingiva is not formed after treatment [27]. This phenomenon can lead to poor esthetic outcomes (e.g., long clinical crown) and is associated with a negative impact on periodontal health (e.g., the formation of gingival recessions) [13].

### Age of patients

The average age of patients referred for orthodontic treatment due to the diagnosis of an impacted central incisor was relatively high (9.5 years), with the oldest patient being 16 years old at initial examination. Considering that upper central incisors erupt at about six years of age, and that a patient with potential impaction should be diagnosed no later than six months after the other incisor has erupted, the vast majority of patients were referred for orthodontic therapy very late. However, these findings correspond to other studies where the initial age of patients was even higher. Chaushu et al. [21, 22] reported a higher average age in their studies (19 and 22 years, respectively). Regular routine check-ups by the family dentist play a crucial role in ensuring appropriate timing of the referral for orthodontic treatment. It remains unknown whether these patients receive orthodontic treatment later due to their own choice or whether they are observed by their dentists for too long before ultimately being referred to an orthodontist.

### Treatment time

The average treatment time in the present study was 22.0 ± 6.7 months. No difference in length of treatment time was found between the two different methods of treatment. Regarding the baseline characteristics, such as cause of impaction, initial angle of the impacted incisors, degree of impaction, and apex maturation stage, the treatment time trended toward being longer in patients with complex impaction or with unfavorable inclination; however, the differences were not statistically significant. This outcome was in accordance with studies by Chaushu et al. [21] and Ho and Liao [26] in which the treatment time was longer for high-positioned and dilacerated teeth. Similar data with respect to patient age were also presented in these studies.

### Etiology of impactions

The cause of impaction was supernumerary tooth in 17 cases and dilaceration of the root in eight cases. The etiology of impaction could not be identified in nine cases. Interestingly, of the patients with root dilaceration, six reported a previous trauma to the deciduous anterior dentition. Stewart [28] found a history of deciduous dentition trauma in 22%, the presence of a cyst in 7%. In 71% of cases, the cause of dilaceration was not established. Chaushu et al. [21] diagnosed obstruction with supernumerary tooth as an etiological factor in 29 cases, dilaceration in 27 cases (14 patients confirmed an earlier trauma) and could not identify the etiology in 4 patients. In dilacerated teeth, an early diagnosis and timely surgical intervention with subsequent active orthodontic traction are crucial for further root development and prevention of worsening of the root dilaceration [29, 30].

### Esthetic and clinical implications

**Fig. 7:**
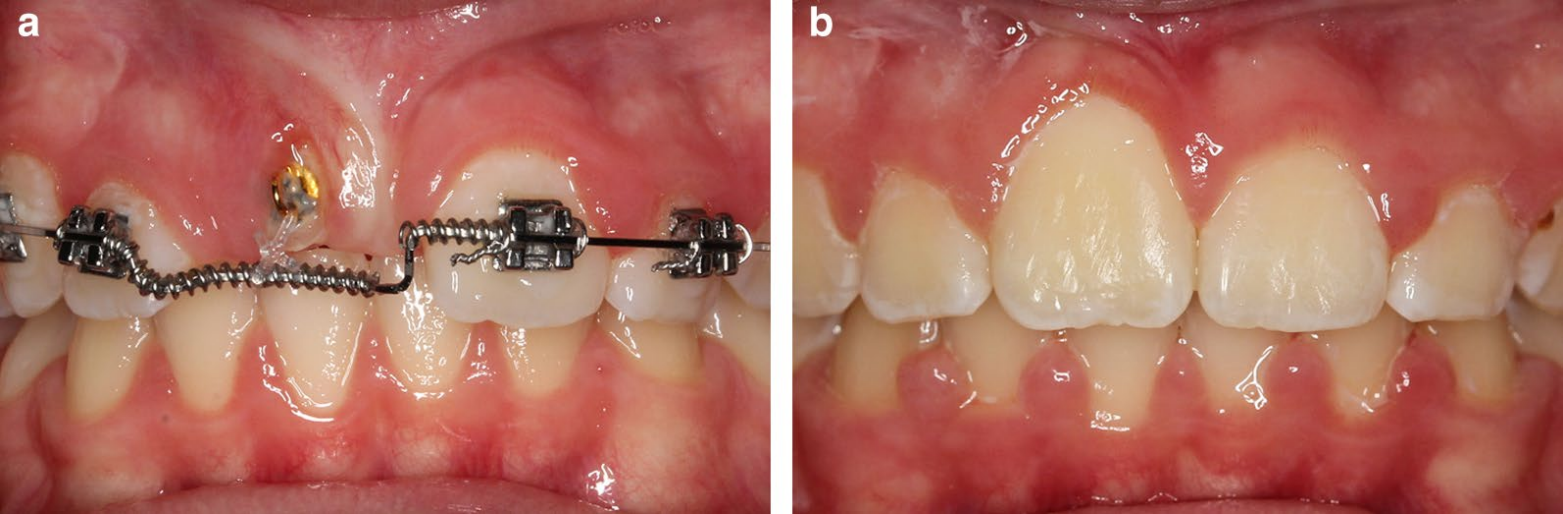
**a** Bonding the orthodontic attachment to the labial surface of the impacted tooth causes rupture of the alveolar mucosa (button-holing); consequently, the attached gingiva fails to appear and the clinical crown is significantly longer (**b**)

Assessment of the attached gingiva showed that the majority of teeth after treatment have either sufficient or satisfactory attached gingiva in patients of both groups. In the closed eruption group, the result was significantly worse when compared to the contralateral reference tooth. However, no significant difference between groups was found between both the groups. Chaushu et al. [23] confirmed that the attached gingiva was established in more patients using the closed eruption surgical method than other surgical methods, while Becker et al. [25] found increased bone loss in the closed eruption method when compared to the contralateral physiologically erupted incisor. Vermette et al. [20] found that after treatment with an apically positioned flap method, the height of the attached gingiva on the labial surface was greater than that of the control tooth. With the closed eruption method, the attached gingiva was narrower. However, with the apically positioned flap method, more bone loss and greater apical shift of the gingival margin were found. Krejčí [31] presented similar results for the apically positioned flap method, but scarring occurred, which is common with this surgical method. For the closed eruption method, a smaller amount of attached gingiva was recorded, but this may be due to the practice of placing the attachment on the vestibular side of the crown of the impacted tooth, causing a rupture of the alveolar ridge mucosa (“button-holing”) (Fig. 7a, b).

Regarding the length of the clinical crown and the associated esthetic outcome, no significant differences were found between groups. Both treatment methods used in this study resulted in clinical crown length comparable to that of contralateral physiologically erupted incisors. This finding is in accordance with studies conducted by Chaushu et al. [21, 23] who compared the esthetic results for the closed eruption technique and apically positioned flap method. The latter showed worse esthetic results; clinical crown length was significantly longer and scarring occurred. Becker et al. [25] also found that the closed eruption method provided esthetic results with minimal negative clinical consequences.

### Limitations of the study

This study included a relatively small sample of patients with impacted maxillary central incisors, which reflects the sparsity of cases of such a rare condition. Yet, to our knowledge, this sample is the largest studied to date. The recruitment period was very long (2002–2017), which could have affected the results as the expertise and skill of the treating specialist likely increased over time. However, the orthodontist and both surgeons followed the treatment protocol in each case, and we believe that the care delivered was consistent throughout the recruitment period.

Another limitation may be the available photographs reviewed in the study. In our opinion, the quality of photographs has not changed significantly over time, as high-quality SLR and later DSLR cameras were used. Also, the initial age of the patients in the sample ranges from 9 to 16 years, which added heterogeneity to each group. However, this age range is similar to groups studied by Chauschu et al., who reported patients aged between 7 and 22 years [21], and 15 to 38 years of age [22] in two studies. Narrowing the age range would provide a more homogenous sample but would also likely decrease the sample size significantly. The age of the patients is always influenced by the timing of patient referral by the treating dentist, and also by the timing of the patient`s decision to seek orthodontic care. Finally, the evaluation of attached gingiva and esthetic outcomes based on categorization may seem subjective; however, the categories were established according to precise measurements. Absolute values of these measurements could not be directly compared due to interindividual differences between patients.

## Conclusion

1. Of the 31 patients with permanent upper central incisor impaction, 16 had impaction on the left and 12 on the right, and bilateral impaction was found in three patients. Impaction was caused by a supernumerary tooth (*n* = 17) or root dilaceration (*n* = 8). Etiology was unknown in nine patients.
2. The average treatment time for all patients was 22.0 ± 6.7 months, with no difference between groups. There was also no difference in treatment time between groups with respect to baseline characteristics such as the orthodontic therapy type, cause of impaction, NS angle, degree of impaction, or apex maturation stage. A positive correlation was found between the treatment time and age in pooled data.
3. The amount of attached gingiva was significantly smaller in the closed eruption group when compared to contralateral reference teeth. However, no significant difference was found between groups.
4. No difference was found between the spontaneous eruption group and the closed eruption group with respect to clinical crown length.

Within the limitations of this study, we conclude that in all impacted teeth treated by modified closed eruption method with palatal traction, the attached gingiva was sufficient, and the assessment of the clinical crown showed no difference when compared to teeth with spontaneous eruption after space opening. Both methods evaluated in this study provided comparable amount of attached gingiva and good esthetic results.

## Data Availability

The data underlying this article are available in the Mendeley Data system under https://doi.org/10.17632/6jryvzmm5c.1.
